# Chronic musculoskeletal pain: traps and pitfalls in classification and management of a major global disease burden

**DOI:** 10.1097/PR9.0000000000001023

**Published:** 2022-08-09

**Authors:** Rolf-Detlef Treede

**Affiliations:** Department of Neurophysiology, Mannheim Center for Translational Neuroscience (MCTN), Heidelberg University, Heidelberg, Germany

## Abstract

Mary-Ann Fitzcharles et al. propose to introduce “regional fibromyalgia” as a new diagnosis. This commentary summarizes why this term is misleading but nonetheless the article may pave the way towards useful concepts for myofascial pains.

**Commentary on:** Fitzcharles MA,Cohen SP, Clauw DJ, Littlejohn G, Usui C, Hauser W. Chronic primary musculoskeletal pain: a new concept of nonstructural regional pain. PAIN Reports 2022;7:e1024.**See also:** Cohen ML. Clarifying “chronic primary musculoskeletal pain”? The waters remain murky. PAIN Reports 2022;7:e1021.

## 1. Why the article by Fitzcharles et al. is worth reading

In this month's collection of Pain Reports, a group of renowned pain experts^9^ present an article on the concept of chronic primary musculoskeletal pain, which I strongly recommend for enlightening reading. They propose a list of clinical characteristics to distinguish between chronic primary and secondary musculoskeletal pain conditions. These terms had been introduced into IASP and WHO terminology in the 11th edition of the *International Classification of Diseases and Related Health Problems* (*ICD-11*).^23^ Although this list is based on expert opinion rather than published evidence, it is a useful contribution for scientific debate on how to improve classification and management of these prevalent chronic pain conditions that together are a major global disease burden.^4^

However, on several accounts the authors fall into traps and pitfalls of terminology: a term that they propose for easier understanding (“regional fibromyalgia”) is rather prone to cause confusion. The attempts to integrate the mechanistic concept of “nociplastic pain” and the older term “myofascial pain” are interesting, but the conclusions are premature without further empirical evidence.

## 2. Distinction between chronic primary vs secondary musculoskeletal pain needs further studies

The concepts of chronic primary vs secondary pain conditions were introduced on transition from *ICD-10* to *ICD-11*.^22,23,32^ This distinction was inspired by the concepts of primary headaches as diseases vs secondary headaches as symptoms of some other disease; similar distinctions between primary and secondary health problems exist for many other medical conditions such as insomnia or hypertension.

Fibromyalgia syndrome (FMS) with chronic widespread and mostly proximal and axial pain is the prototypical chronic primary pain condition.^22^ Complex regional pain syndrome (CRPS) shares features with FMS,^19^ but the pain is regional and predominantly distal; it has recently been reclassified from autonomic nervous system disease to chronic primary pain.^35^ There are established diagnostic criteria for both FMS and CRPS.^11,34^

The published criteria for clinical distinction between chronic primary musculoskeletal pain (MG30.02) vs chronic secondary musculoskeletal pain (MG30.3) have not undergone rigorous empirical testing so far.^22^ The same is true for chronic primary visceral pain (MG30.00) vs chronic secondary visceral pain (MG30.4). Early international field trials by IASP and WHO suggest that this distinction is intuitively possible for both pain specialists and primary care physicians.^2,3^ The list published by Fitzcharles et al.^9^ provides a good structure for an empirical validation.

## 3. Why the proposed term “regional fibromyalgia” is likely to cause confusion

In *ICD-11*, the chronic primary pain conditions include a regional pain syndrome (CRPS, MG30.04) and chronic widespread pain (including FMS, MG30.01) as 2 separate entities. Widespread pain (in 4 of 5 body regions)^5^ is a prerequisite for making the diagnosis of fibromyalgia,^34^ whereas regional pain is part of the CRPS definition. Thus, “regional fibromyalgia” would be a contradiction in terms.

For regional pains, CRPS (MG30.04) may sound like the appropriate diagnosis, but the Budapest criteria for sensory, autonomic, and motor signs have provided evidence-based positive identification criteria^11^ that must be fulfilled for this diagnosis. This leaves “chronic primary musculoskeletal pain” (MG30.02) as the default code for the patients who Fitzcharles et al.^9^ have in mind. According to the WHO website,^35^ primary musculoskeletal pain includes:

 (1) Chronic primary low back pain
(2) Chronic primary cervical pain
(3) Chronic primary thoracic pain
(4) Chronic primary limb pain

This is a classification by region, but there is no evidence whether or not any or all of the primary musculoskeletal pain conditions are regional pains; they might be highly localized pains within these 4 body regions.

As it happens, empirical work on a precise definition of the term “widespread pain” has been published,^5^ but a similar critical empirical analysis for the “term regional” pain is missing. This is a major gap in current pain literature that needs to be filled; but the proposed term (regional fibromyalgia) fails to provide a solution.

## 4. A renaissance for the term “myofascial pain”?

The term “musculoskeletal” refers to important elements of the locomotor system; it may be time to deconstruct it into its constituents. First, the locomotor system only functions appropriately when controlled by the somatomotor and somatosensory nervous systems; this is reflected in *ICD-11* in the new entity “Chronic secondary musculoskeletal pain due to disease of the nervous system” (MG30.32).^23,35^ Second, hard tissues (bones or joints) and soft tissues (muscles or fasciae) of the locomotor system need to be assessed by different diagnostic techniques; this may be a reason why they are sometimes called “specific” (hard tissue) vs “nonspecific” (soft tissue).

Fitzcharles et al.^9^ discuss similarities and differences between an old concept called “myofascial pain syndromes” (MPS)^29^ and the new concept of chronic primary musculoskeletal pain. The concept of MPS is closely linked to the concept of trigger points, both as a diagnostic sign and a therapeutic target.^25^ Despite their rather similar wording, trigger points are not the same as tender points,^33^ and MPS are not the same as FMS. However, both have in common that there are no radiological signs on imaging of hard tissues of the locomotor system.

Biology of muscles and fasciae has received less attention than pathology of vertebrae, joints, and disks of the spinal column. However, recent progress on the neurobiology of low back muscle and fascia innervation^14^ and on the psychophysics of muscle and fascia pain^26,28^ has led to novel rodent models of myofascial pain. These models mimic repeated mild injury by i.m. injections of small amounts of nerve growth factor that do not cause pain in humans.^8,12^ Central sensitization of spinal neurons through neuron–glia interactions and neuroinflammation is a key mechanism in these models^13,18,36^ and may reflect spinal long-term potentiation.^17^ These models are exacerbated by stress^27^ and are alleviated by active or passive physiotherapy.^7,24^

Does this make myofascial pain a chronic primary pain or a chronic secondary pain? Fitzcharles et al.^9^ seem to propose that it is secondary. However, I would argue that failure of previous attempts to make the concept of myofascial pain generally accepted may have been because it was described as another chronic secondary pain condition wherein the muscle or fascia abnormalities would fully explain the pain syndrome. The new concept of chronic primary musculoskeletal pain does not exclude somatic factors (muscle or fascia), hence what used to be called chronic myofascial pain may better be classified as chronic primary musculoskeletal pain. To raise empirical evidence either way, a third column “myofascial pain syndromes” should be added to Table 1 in Ref. 9. The table will then allow readers to judge whether myofascial pain is more similar to chronic primary or secondary musculoskeletal pain.

This expanded comparison should pave the way towards replacing the term “nonspecific low back pain” by “chronic primary low back pain” combined with progress on better understanding of its pathophysiology. The rodent models suggest that with such better understanding there will be more “biology” in the biopsychosocial model of low back pain, in this case neurobiology of muscle and fascia innervation and central nervous system signal processing.

## 5. Two or 3 mechanistic descriptors

Chronic pain conditions that are not supported by objective tissue damage are a challenge to understand, diagnose, and manage. Several years ago, they were often labelled “neuropathic” to indicate altered signal processing in the nervous system, but this terminology led to conflation of these conditions with lesions or diseases of the somatosensory system that have neuronal tissue damage as the underlying disease of which they are a symptom.^31^ With the distinction between nociceptive and neuropathic pain in IASP terminology,^20^ positive identification criteria became available for each of them as well as for their combination (both = “mixed pain”); chronic pain conditions without damage to neural or nonneural tissue were left without a clear designation (neither = “idiopathic”).^10^

This situation motivated a group of pain experts to develop a third mechanistic descriptor “nociplastic pain.”^16^ The grading system for its diagnosis^15^ starts by establishing that the pain is neither nociceptive nor neuropathic (equivalent to the designation as idiopathic) and then calls for clinical evidence for central sensitization. Central sensitization, however, is not a unitary phenomenon. It occurs at least at 3 levels (spinal, brainstem, and cortical)^30^ and is basically a set of normal physiological processes.^21^ Spinal long-term potentiation is involved in secondary hyperalgesia that occurs with nearly all acute nociceptive pains. Brainstem controls are activated in both nociceptive and neuropathic pain conditions^1^ and so are cortical sensitization mechanisms.^37^ Thus empirical studies are needed that quantify the extent of the subtypes of central sensitization across the entire range of acute, chronic primary and chronic secondary pains. It is premature to use “nociplastic pain” as synonym for mechanisms of chronic primary pain conditions.^6^

## 6. Conclusion: a fresh look at the biopsychosocial model of musculoskeletal pain

Biology of muscles and fasciae has traditionally been neglected over pathology of injured vertebrae, joints, and disks of the spinal column. Because muscle or fascia damage does not show on conventional X-ray or computed tomography, it is likely that many patients with so-called nonspecific low back pain actually suffer from such soft tissue damage. The proposed term “regional fibromyalgia”^9^ is unlikely to be helpful because it muddles the distinction between widespread and regional pains, but a fresh look at myofascial pain syndromes and to what extent they are primary or secondary pains will be useful for a better distinction between soft tissue and hard tissue involvement. Thus, this article is a good starting point to refine concepts and design pivotal diagnostic trials in the vast field of musculoskeletal pain conditions.

## Disclosures

The author has no conflict of interest to declare.
